# Superior efficacy of co-treatment with the dual PI3K/mTOR inhibitor BEZ235 and histone deacetylase inhibitor Trichostatin A against NSCLC

**DOI:** 10.18632/oncotarget.11109

**Published:** 2016-08-06

**Authors:** Junjie Piao, Liyan Chen, Taihao Quan, Longshan Li, Chunji Quan, Yingshi Piao, Tiefeng Jin, Zhenhua Lin

**Affiliations:** ^1^ Department of Pathology & Cancer Research Center, Yanbian University Medical College, Yanji 133002, China; ^2^ Key Laboratory of Natural Resources of Changbai Mountain & Functional Molecules (Yanbian University), Ministry of Education, Yanji 133002, China; ^3^ Department of Dermatology, University of Michigan Medical School, Michigan 48109-5609, USA; ^4^ Department of Radiation Oncology, Simmons Comprehensive Cancer Center, University of Texas Southwestern Medical Center, Dallas, Texas 75390-8807, USA

**Keywords:** non-small-cell lung cancer, TSA, BEZ235, epithelial-mesenchymal transition

## Abstract

Non-small-cell lung cancer (NSCLC) is the leading cause of cancer-related death worldwide. NSCLC development and progression have recently been correlated with the heightened activation of histone deacetylases (HDACs) and PI3K/Akt signaling pathways. Targeted inhibition of these proteins is promising approach for the development of novel therapeutic strategies to treat patients with advanced NSCLC. For this reason, we combined a dual PI3K and mTOR inhibitor, BEZ235 with the HDAC inhibitor Trichostatin A (TSA), to determine their combined effects on human NSCLC. In this study, we initially discovered that co-treatment with BEZ235 and TSA showed a synergistic effect on inhibition of NSCLC cell proliferation and induction of apoptosis. The combination treatment also synergistically suppressed NSCLC migration, invasion and the NSCLC epithelial-mesenchymal transition (EMT) *in vitro*. The synergistic effect was also evidenced by declines in xenograft growth and metastasis rates and in ki-67 protein expression *in vivo*. Together, these results indicated that BEZ235 and TSA combination treatment significantly increased anti-tumor activities compared with BEZ235 and TSA alone, supporting a further evaluation of combination treatment for NSCLC.

## INTRODUCTION

Lung cancer is one of the most commonly diagnosed cancers worldwide [1]. NSCLC is the major histological form of epithelial lung cancer, and it remains the principle cause of cancer-related death, accounting for more than one million deaths per year [2, 3]. Recent advances in molecular diagnosis and targeted therapies have providing opportunities to select lung cancer patients based on their individual molecular characteristics to receive personalized targeted therapies that translate into clinically meaningful benefits [4]. For example, EGFR-TKI based targeted therapy generates a dramatic response in patients with advanced EGFR-mutation positive NSCLC [5, 6]. Unfortunately, most current therapeutic approaches are limited by drug resistance and subsequent disease relapse, as evidenced by the median overall survival rate of 25.5 months [7, 8]. Increasing evidences has suggested that acquired drug resistance limits the long-term effectiveness of EGFR-TKIs. Thus, a new therapeutic strategy is necessary for NSCLC treatment.

Phosphatidylinositol 3-kinase (PI3K)/ protein kinase B (Akt)/ mammalian target of rapamycin (mTOR) signaling are a major survival pathway. Its abnormal activation, primarily caused by K-ras gene mutations in NSCLC, is frequently involved in the development and progression of NSCLC [9, 10]. BEZ235, a dual class I PI3K/mTOR inhibitor, is currently under evaluation in phase I/II clinical trials. BEZ235 reverses the hyperactivation of the PI3K/Akt/mTOR pathway, resulting in antitumor activities in cancers of various origins [11–13]. Additionally, studies have shown that together with other chemotherapeutic agents, including Everolimus and Selumetinib, BEZ235 exhibits synergistic antitumor effects on NSCLC cells [14, 15]. These data suggest that the potential clinical activities of BEZ235 can be combined with other chemotherapeutic agents to synergistically kill cancer cells.

HDACs catalyze the deacetylation of histones to promote a compact chromatin configuration that restricts access to DNA by transcription factors resulting in repressed gene expression. HDACs are up-regulated in various cancers, including gastric, breast and lung cancers [16, 17]. The heightened HDACs levels and subsequent histone acetylation reversals have made them excellent targets for cancer therapy. TSA, an HDAC inhibitor (HDACi), has a broad spectrum of epigenetic activities, and selectively inhibits the class I and II mammalian HDAC families [18–20]. Recently, TSA was shown to modulate the expression of several genes that regulate apoptosis, angiogenesis, cell cycle and differentiation [21, 22]. Studies have also suggested that the HDAC inhibitor can sensitize cancer cells to chemotherapy [23–25]. Thus, we hypothesized that the combined use of BEZ235 and TSA would have a synergistic therapeutic effect on NSCLC.

In this study, we determined the combined effects of BEZ235 and TSA against human NSCLC cells. The results demonstrate that these two agents cause synergistic growth arrest and cooperatively induce apoptosis. Synergistic suppression of migration, invasion and EMT progression *in vitro* is also observed. Moreover, xenograft studies revealed that these two drugs cooperated to suppress tumor growth and metastasis, and to induce tumor necrosis *in vivo*. These results provided support for the combination of BEZ235 and TSA in the treatment of NSCLC.

## RESULTS

### Combination treatment with BEZ235 followed by TSA leads to synergistic cytotoxic effect on A549 and H460 cells

**Figure 1:**
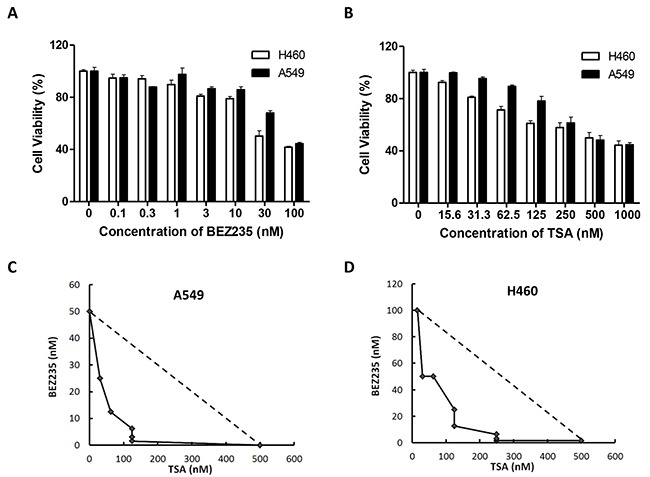
Combination treatment with BEZ235 followed by TSA leads to synergistic cytotoxic effect on A549 and H460 cells **A-B.** A549 and H460 cells were treated with the indicated concentrations of BEZ235 or TSA for 24 h. Viable cells were measured using the MTT assays. **C-D.** Isobologram analyses were performed, and CI values were calculated. CI values less than 1.0 indicate the synergistic interaction of the two agents in the combination.

To valuate cell proliferation, A549 and H460 cells were exposed to increasing doses of either BEZ235 or TSA, and the treatment effects were assessed using the MTT assay. Both BEZ235 and TSA significantly reduced A549 and H460 cell viability. The IC_50_ values for BEZ235 were 79.63nM and 31.81nM for A549 and H460 cells, respectively, and those for TSA were 339.72nM and 482.31nM, respectively (Figure 1A-1B). Thus, we set the indicated BEZ235 and TSA concentrations to 50nM and 250nM, respectively. We then tested the combination of BEZ235 and TSA for possible synergistic killing of A549 and H460 cells. The isobologram analysis indicated that the combination treatment effect was highly synergistic in both A549 (combination index, CI=0.46) and H460 (CI=0.71) cells (Figure 1C-1D), where the synergy was higher in A549 than in H460 cells.

### Co-treatment with BEZ235 and TSA synergistically induces apoptosis of A549 and H460 cells

**Figure 2:**
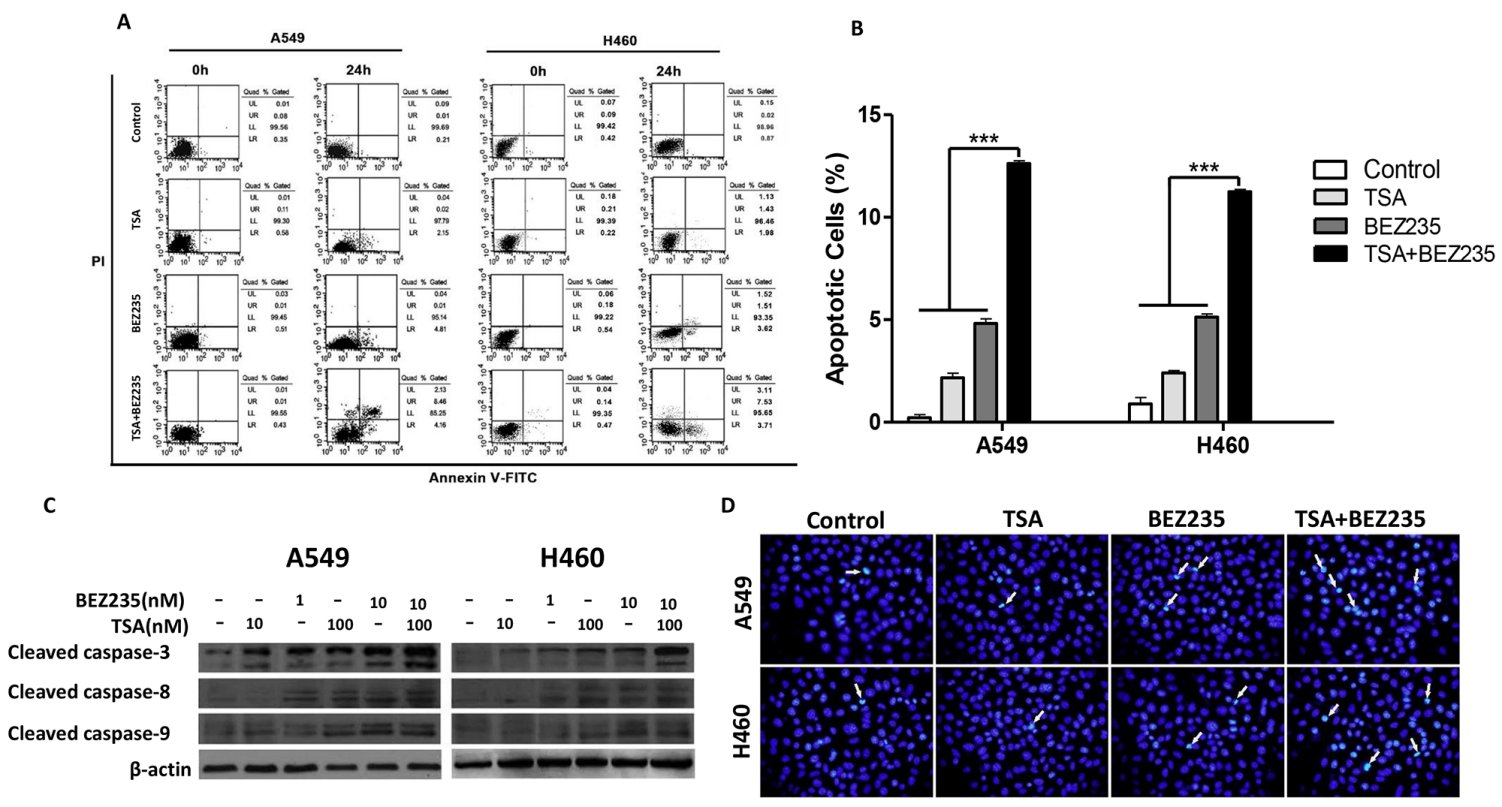
Combination treatment with BEZ235 followed by TSA induces apoptosis in A549 and H460 cells **A-B.** A549 and H460 cells were exposed to the indicated concentration of BEZ235 and/or TSA for 24 h, and the cells were stained with Annexin V-FITC/PI. Apoptotic cells were analyzed by flow cytometry.(***p<0.001). **C.** Protein levels of cleaved caspase-3, -8 and -9 were determined by western blot. **D.** Cells were stained with Hoechst33342 and analyzed by fluorescence microscopy. Arrow point identifies apoptotic nuclei.

We determined the effects of the combination treatment on apoptotic induction. Annexin V-FITC/PI staining followed by a flow cytometric analysis was performed on A549 and H460 cells that had been treated with BEZ235 and TSA for 24 hours. As shown in Figure 2A-2B, the combination treatment significantly increased the number of apoptotic cells compared with individual BEZ235 or TSA treatment (*p*<0.001).

Caspases play pivotal roles in cell apoptosis, and several chemotherapeutic agents kill cancer cells through caspase-dependent pathways. We examined the effect of BEZ235 and TSA on caspase activity by western blot to determine whether caspase activation occurs during BEZ235- and TSA-induced apoptosis. The results showed that co-treatment with BEZ235 and TSA increased the expression levels of cleaved caspase-3, -8, -9 (Figure 2C). Additionally, Hoechst33342 staining showed that co-treated A549 and H460 cells exhibited significantly more cells with condensed and fragmented nuclei than those treated with either BEZ235 or TSA alone (Figure 2D). These results suggest that co-treatment with BEZ235 and TSA synergistically induce apoptosis in A549 and H460 cells.

### BEZ235 suppresses p-Akt, p-70S6K, and p-4EBP1, and TSA mainly suppresses HDAC1, HDAC2 and HDAC6 expression

**Figure 3:**
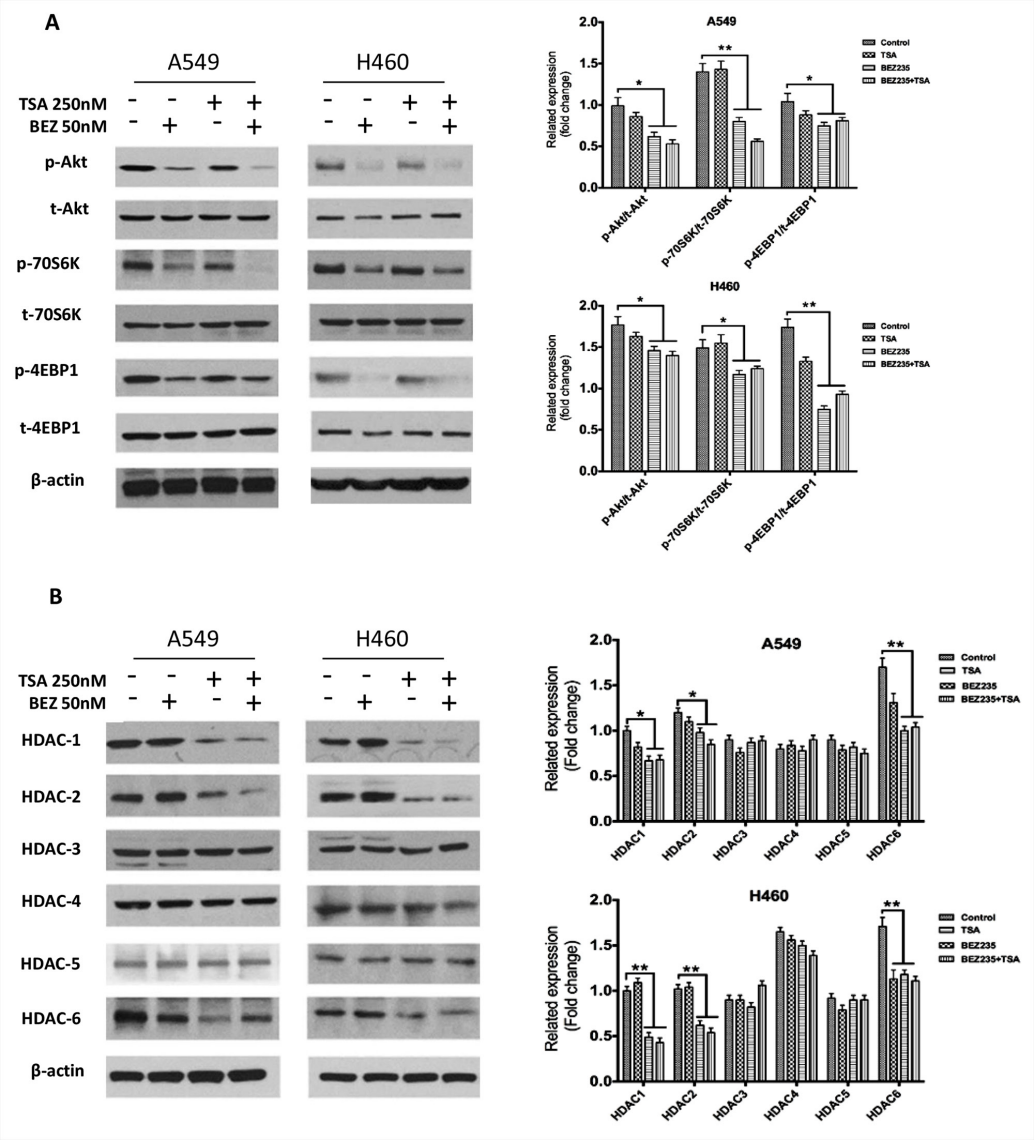
BEZ235 suppresses p-Akt, p-70S6K, and p-4EBP1, whereas TSA mainly suppresses HDAC1/2/6 in A549 and H460 cells A549 and H460 cells were treated with the indicated concentrations of BEZ235 and/or TSA for 24h. Total cell lysates were subsequently prepared, and western blot analyses were performed for t-Akt, p-Akt (Ser473), t-70S6K, p-70S6K (Thr389), t-4EBP1, p-4EBP1 (Thr37/46), HDAC1, HDAC2, HDAC3, HDAC4, HDAC5 and HDAC6. The β-actin expression level in the lysates served as the loading control.(*p<0.05, **p<0.01).

HDAC expression and the activation status of several proteins in the PI3K/Akt/mTOR pathway were assessed in A549 and H460 cells by western blot. A549 and H460 cells were treated with DMSO, BEZ235, TSA or BEZ235 and TSA, and the protein levels were measured by western blot. The results showed that BEZ235 inhibited PI3K/Akt/mTOR pathway activation, indicated by the phosphorylation status of Akt, 4EBP1 and 70S6K, respectively (Figure 3A). Conversely, TSA reduced HDAC1, HDAC2 and HDAC6 levels, but not Akt and p-Akt (Figure 3B).

We also demonstrated the co-treatment effects on the PI3K/Akt/mTOR pathway and HDACs expression in A549 and H460 cells. As shown in Figure 3, co-treatment with BEZ235 and TSA was more effective on reducing p-Akt (Ser473) and p-70S6K (Thr389) than treatment with each agent alone. However, the combination treatment-mediated declines in the HDACs and p-4EBP1 (Thr37/46) levels were similar to those caused by BEZ235 or TSA alone.

### BEZ235 and TSA synergistically inhibit the migration, proliferation and invasion ability of A549 cells

**Figure 4:**
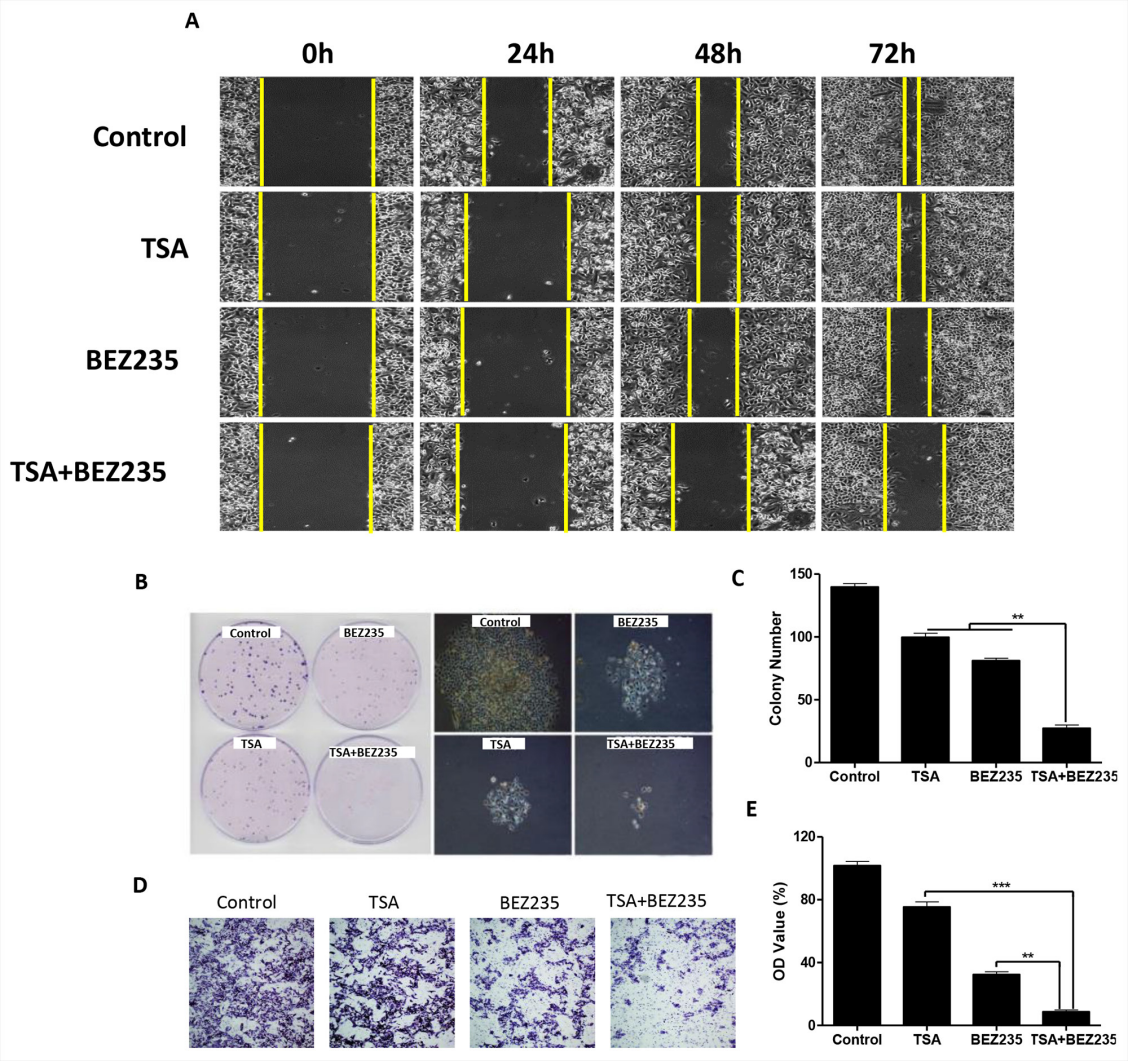
Co-treatment with BEZ235 and TSA synergistically inhibited A549 cancer cell proliferation, migration and invasion **A.** A549 cancer cells were treated with the indicated concentrations of BEZ235 and/or TSA for 24 h. Photographs were taken at 0h, 24h, 48h and 72h, after the wound was made. The normalized wound area was calculated using a software-base method. **B-C.** A549 cells were seeded, and then treated with the indicated concentrations of BEZ235 and/or TSA for 24h, and morphological changes were visualized by phase-contrast microscopy. The cells were stained and the colonies were photographed. The number of colonies was determined by microscope. **D-E.** A549 cells were seeded and treated with the indicated concentrations of BEZ235 and/or TSA for 24h, and the number of invasive cells was determined using a transwell matrix penetration assay (with matrigel).(**p<0.01, ***p<0.001).

To determine the effect on cell migration, proliferation and invasion, several functional studies were performed. Wound healing assays showed that the migratory ability of A549 cells was significantly inhibited in the co-treatment group, compared with the groups treated with DMSO, BEZ235, or TSA (Figure 4A). Colony formation assays were performed to verify the synergistic effect on cell proliferation. The results showed that co-treatment with BEZ235 and TSA significantly inhibited A549 colony formation compared with the other groups (Figure 4B-4C) (*p*<0.01). Although treatments with BEZ235 or TSA reduced the invasion of A549, the reduction induced by the combination treatment was remarkably stronger (Figure 4D-4E). These functional studies highlight the synergistically inhibitory effects of BEZ235 and TSA on NSCLC *in vitro*.

### BEZ235 and TSA synergistically suppress the EMT progression of A549 and H460 lung cancer cells

**Figure 5:**
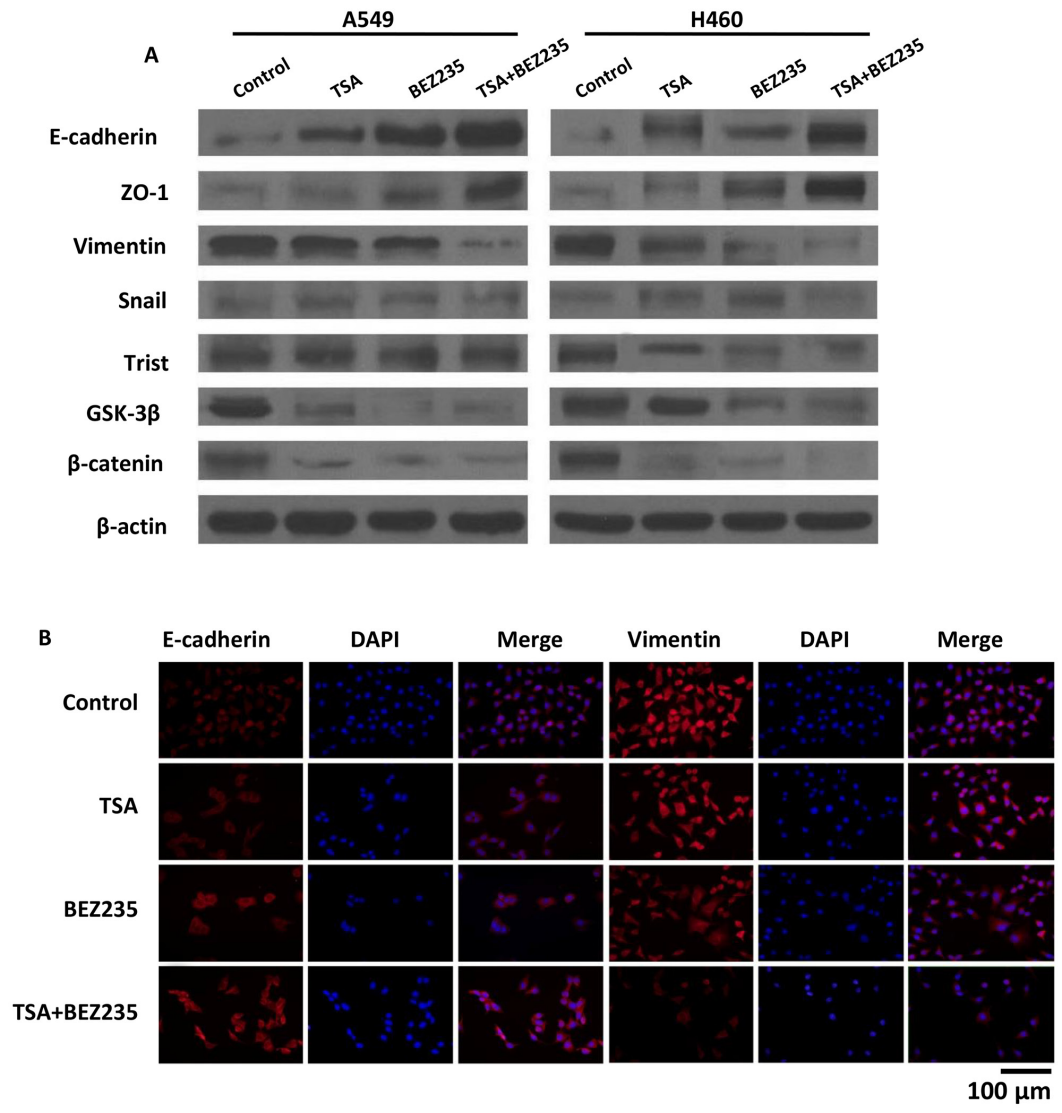
BEZ235 and TSA synergistically suppressed the EMT progression of A549 cells **A.** A549 and H460 cells were treated with the indicated concentration of BEZ235 and/or TSA for 24 h. Then, the total cell lysates were prepared and western blot analyses were performed for EMT-associated markers (E-cadherin, Snail, Vimentin, ZO-1, Twist, β-catenin and GSK-3β). The β-actin expression level in the lysates served as the loading control. **B.** EMT-associated markers (E-cadherin, Vimentin) were visualized by IF.

During the progression of a malignancy, the carcinoma cells that undergo EMT lose their epithelial characteristics and acquire migratory properties are critical for tumor dissemination [26]. We examined EMT-associated protein expression by western blot and immunofluorescence (IF). The results revealed that the co-treatment groups showed lower expression levels of mesenchymal-related proteins, including Vimentin, Twist, GSK-3β, and β-catenin, than the other treatment groups. Additionally, lower expression levels of the epithelia cell-related protein E-cadherin and ZO-1 were observed by western blot (Figure 5A). The altered expression levels of Vimentin and E-cadherin were also observed in A549 cells by IF (Figure 5B). These results indicate that co-treatment with BEZ235 and TSA synergistically suppresses EMT in A549 and H460 cells.

### The BEZ235 and TSA combination synergistically suppresses tumor growth and metastasis inA549 xenograft models

**Figure 6:**
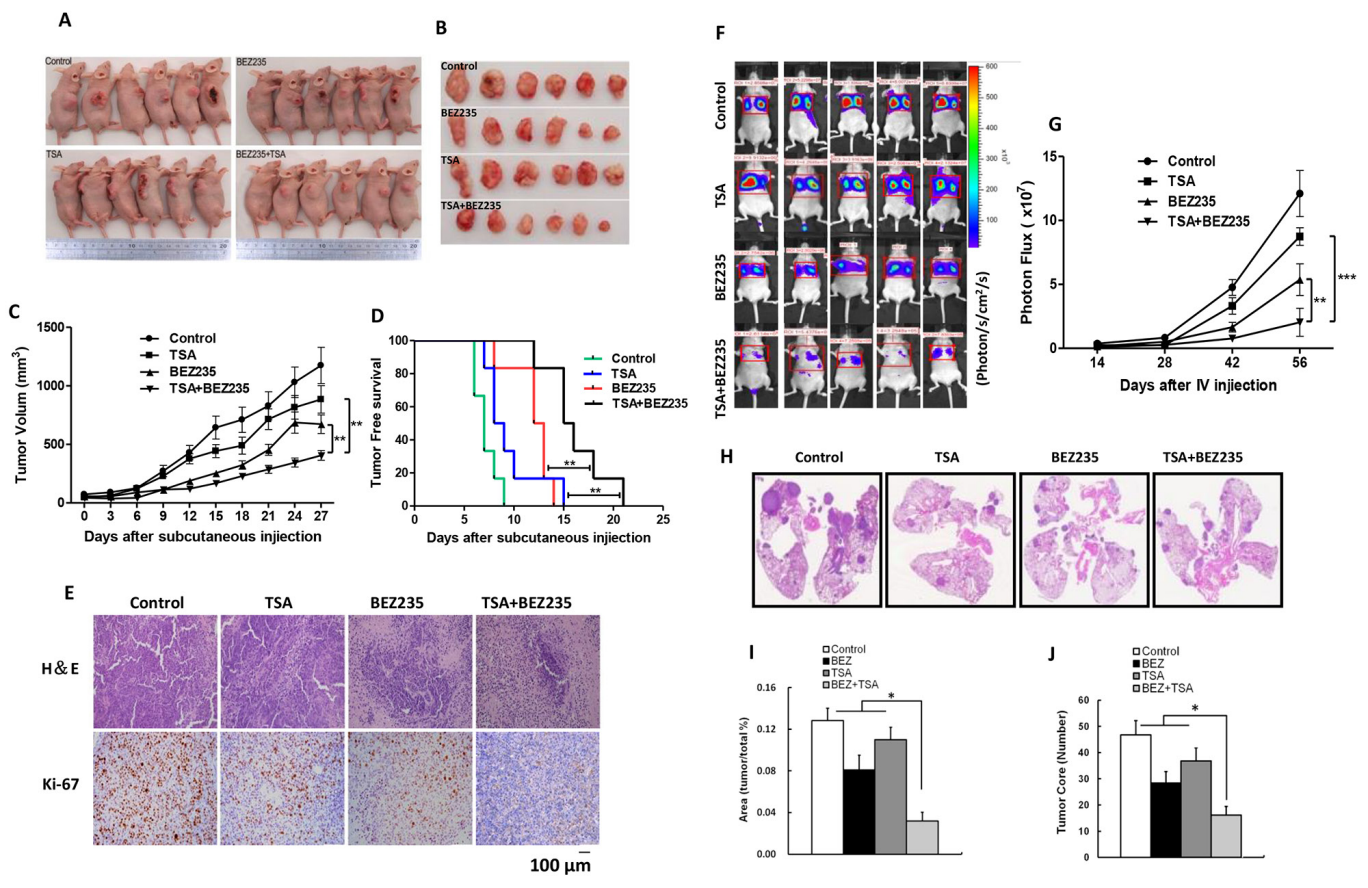
BEZ235 and TSA synergistically suppressed the growth and metastasis of A549 cells *in vivo* **A-C.** The mice were sacrificed 27 days after drug treatment initiation, and the tumors were removed. The tumor volumes were measured every 3 days during the treatment. **D.** Kaplan-Meier survival curve of nude mice implanted with A549 tumors in the each groups. **E.** Slides containing the tumor specimens were examined for Ki-67 positive or by and H&E staining. **F-G.** A549 cells were injected into the tail vein of nude mice. 8 weeks after drug treatment initiation, whole-body imaging of mice was performed using IVIS. **H-J.** The lungs from these mice were excised from the surrounding tissue for H&E staining and analysis of the tumor area and tumor core.(*p<0.05, **p<0.01, ***p<0.001).

We determined the effects of BEZ235 and TSA on tumor growth using established xenograft generated by subcutaneous dorsal implantations of A549 cells into nude mice. As shown in Figure 6A-6C, four weeks treatment with either BEZ235 or TSA alone significantly reduced tumor growth compared with the control, resulting in lower mean of A549 tumor volume (*p*<0.01). Additionally, combination treatment prevented tumor development more effectively than either single treatment, resulting in superior tumor free survival of co-treated mice (Figures 6D). To further investigate the effects of these drugs treatments *in vivo*, tumors from 3 mice in each group were analyzed by H&E and immunohistochemical (IHC) staining. Interestingly, IHC staining showed lower expression of Ki-67 in the co-treatment group than in the other groups. H&E staining also revealed increasing necrosis in the drug-treated tumors groups compared with the control group; necrosis was especially high for the combination group (Figure 6E). To investigate whether the synergistic effect of the two drugs on suppressing metastasis *in vivo*, we injected A549 cells into the tail veins of nude mice, and treated the mice with BEZ235 and TSA, either alone or in combination. The results showed that co-treated mice developed fewer tumor nodules in the lungs than the other groups (Figure 6F-6J).

## DISCUSSION

The PI3K/Akt/mTOR pathway is often dysregulated in cancer due to mutations, deletions, amplifications, methylation changes and post-translational modifications [27]. Activation of this pathway in NSCLC promotes cell proliferation, growth, survival and an aggressive phenotype, and also confers therapeutic resistance to chemotherapeutic agents [28, 29]. HDAC-mediated epigenetic modifications are essential for regulating gene expression. Cancer cells acquire pathological epigenetic modifications that promote gene expression patterns that facilitate and sustain tumorigenesis, and these modifications are associated with cancer chemo-resistance. A genetic profile of alterations in human NSCLC has shown that simultaneous increases in PI3K/Akt/mTOR pathway activity and in dysregulated HDAC expression are common [30]. These alterations promote the aggressive phenotype and confer chemoresistance, due to co-regulation of important downstream targets by these two pathways. These observations indicate that co-treatment with inhibitors of the two pathways may be a new therapeutic strategy for NSCLC treatment. In our study, we showed that co-treatment with BEZ235 and TSA caused significant NSCLC cell growth arrest both *in vitro* and *in vivo*, with effects ranging from additive to synergistic. These findings are consistent with previous reports that have documented effectively inhibition of BEZ235 and TSA combination treatment on proliferation and colony formation in pancreatic ductal carcinoma cells [31]. In addition, Ellis L *et al.* reported that combined treatment of HDAC inhibitor, PAN with BEZ235 synergistically inhibited pancreatic cancer cell growth [32]. Moreover, *in vivo* xenograft study here also demonstrated that BEZ235 and TSA inhibited NSCLC growth when combined. We concluded that BEZ235 and TSA synergistically inhibited NSCLC growth both *in vitro* and *in vivo*.

As well known, the detection of active caspases in cells and tissues is an important method for measuring apoptosis induced by a wide variety of apoptotic signals [33]. Here, cleaved caspase-3, -8 and -9 expression were synergistically up-regulated, as expected, in the co-treated group, suggesting that BEZ235 and TSA synergistically inhibited NSCLC growth by inducing apoptotic cell death. Similarly, Hajji N *et al*. reported that combination of the HDAC inhibitor, TSA, and topoisomerase II inhibitor, etoposide induces caspase-mediated apoptotic cell death in NSCLC cells [34].

In agreement with previous reports [14], we showed that BEZ235 strongly reduced p-Akt (Ser473), p-4EBP1 (Thr37/46) and p-70S6K (Thr389) levels, and that TSA reduced HDAC1, HDAC2 and HDAC6 expression. We also verified that co-treatment with BEZ235 and TSA caused a synergistic reduction of p-Akt (Ser473), and p-70S6K (Thr389) levels. As a downstream molecule of PIP_3_ in the PI3K pathway, 70S6k plays a pivotal role in cell metabolism and in cancer progression [35]. Zoncu R *et al*. reported that phosphorylation of 70S6K is a key regulator of the proliferation and survival of cancer cells [36]. Together with our results, we confirmed that combined treatment with BEZ235 and TSA could more effectively inhibit the growth and survival of NSCLC cancer cells through dephosphorylation of 70S6K.

EMT is the process by which epithelial cells acquire fibroblast-like properties, reduce their adhesions, and become migratory and invasive. These characteristics are important for cancer cell metastasis [37]. Moreover, EMT induced Tumor cell dissemination and drug ineffectiveness in cancer therapy have been intensively studied [38, 39]. Recent studies have suggested that inhibition of the PI3K/Akt pathway and HDACs expression suppresses EMT progression. Wang X *et al*. reported that TSA reverses EMT in colorectal cancer and prostate cancer cells [40]. Lin G *et al*. also reported that BEZ235 prevents epithelial-mesenchymal transition induced by hypoxia and TGF-β1 [41]. In current study, we validated BEZ235 and TSA as potent EMT modulators that synergistically suppressed EMT in NSCLC cells *in vitro*, and suppressed NSCLC metastasis *in vivo*. Taken together, these results suggest that the BEZ235 and TSA combination can effectively suppress NSCLC malignant transformation by driving cancer cells towards an epithelial state. However, the mechanisms driving EMT warrant further elucidation.

Gammoh N *et al*. reported that PI3K/Akt/mTOR pathway inactivation and HDAC inhibitor treatments induce autophagy [42], which is associated with autophagic cell death. Therefore, it is conceivable that co-treatment with BEZ235 and TSA may induce autophagy, leading to the engagement of an autophagic cell-death mechanism and tumor growth inhibition. Moreover, reports have also shown that PI3K/Akt pathway inactivation and HDAC inhibitor treatments suppress tumor angiogenesis [43, 44]. Further studies are needed to explore the effects of BEZ235 and TSA on NSCLC angiogenesis and autophagy.

**Figure 7:**
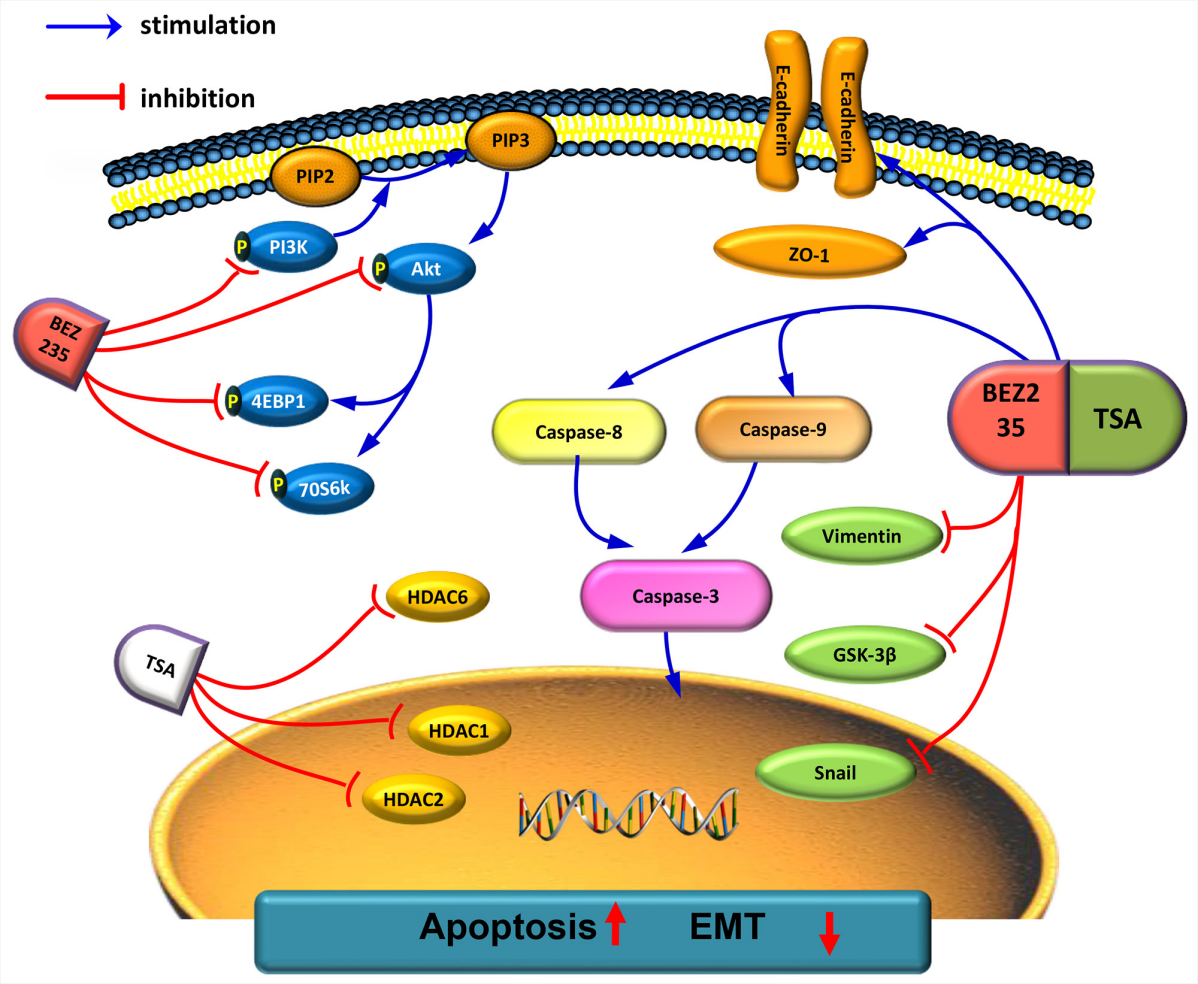
Schematic representation of combination effect of BEZ235/TSA against NSCLC Combination treatment of BEZ235 with TSA induced apoptosis and inhibits EMT formation to against NSCLC malignant process.

In conclusion, we used functional studies to demonstrate that combined treatment with BEZ235 and TSA synergistically inhibits proliferation and induces apoptosis in NSCLC cells, and these activities may occur through inactivating PI3K/Akt/mTOR pathways. Additionally, our data suggested that combining BEZ235 with TSA synergistically suppresses NSCLC migration, invasion and EMT *in vitro*. Furthermore, co-treatment with BEZ235 and TSA synergistically suppresses tumor growth and metastasis *in vivo* (Figure 7). These results highlight the need to clinically develop this therapeutic strategy to treat patients with advanced lung cancer.

## MATERIALS AND METHODS

### Cell culture

Yanbian University Cancer Research Center supplied the A549 and H460 human lung cancer cell lines. All cell lines were cultured at 37°C, 5% CO_2_in RPMI-1640 medium supplemented with 10% FBS and 1% penicillin/streptomycin.

### Cell viability assay

One thousand cells were seeded into 96-well plates to adhere overnight. Cells were then incubated with indicated treatments for 24h, followed by the addition of 5mg/ml MTT (Sigma, St. Louis, MO). After incubation at 37°C for 4h, the supernatants were removed. 200μl of DMSO were added, and the absorbance value (OD) at 570nm was measured using an ELISA reader system (TECAN-infinite M200 pro, Mannedorf, Switzerland). The combination index (CI) was obtained using commercially available software (Calcusyn; Biosoft, Ferguson, MO).

### Colony formation assay

Two hundred cells were seeded into 100 mm dishes in complete RPMI-1640, cultured for 24h, and treated with TSA, BEZ235, or TSA and BEZ235 for 24h. The cells were then washed twice and cultured for up to 3 weeks. Colonies were visualized by Coomassie blue staining and counted according a defined colony size.

### Wound healing assay

Cells were seeded in 6-well plates to adhere overnight. The cell monolayer was then scraped with a pipette tip to form a wound. Cells were subsequently treated with indicated concentrations of TSA, BEZ235, or TSA plus BEZ235. Wound images were photographed at 0h, 24h, 48h and 72h after the cell monolayer was scraped.

### Transwell assay

10^6^/ml Cells in 200 μl serum-free RPMI-1640 were seeded into the upper chamber of the BioCoat™ Invasion Chambers (BD, USA), which contained a matrigel coated polycarbonate transwell filter. Cells were incubated at 37°C for 24 h and removed from inside the upper chamber with cotton swabs. The cells invaded through the transwell filter to the lower membrane surface were fixed in 1% paraformaldehyde and stained with 0.1% crystal violet. Glacial acetic acid was used to dissolve crystal violet, and the OD values were measured.

### Flow cytometry

Cells were cultured in 6-well plates, and subsequently treated with BEZ235 or TSA alone or in combination. Cells were then harvested, washed and re-suspended in binding buffer, and labeled with Annexin V-FITC and PI for 20min by using an apoptosis detection kit (BD). Fluorescence was measured by flow cytometry (BD).

### Hoechst33342 staining

Cells were seeded at 1×10^4^ cells/ml and cultured for 2 to 4 days until they were 80 to 90% confluence. The cells were then fixed for 10min at room temperature (RT) with 3% paraformaldehyde, followed by staining with 1mg/ml Hoechst33342 at RT for 5min. The cells were then washed twice with PBS, examined and immediately photographed under a fluorescence microscope.

### Protein extraction and western blot

Protein was extracted using RIPA buffer (Sigma, St. Louis, MO). The protein concentration was quantified using the Bradford Protein Assay Kit (Pierce Biotechnology, Rockford, IL). Cell lysates were resolved by SDS-PAGE and transferred to a PVDF membrane (Millipore, Eschbom, Germany). The membrane was then blocked using 5% non-fat milk and incubated with the indicated primary antibody at 4°C overnight, followed by incubation with a secondary antibody (Cwbiotech, Beijing, China) for 2 h at RT. Protein expression was detected using the ECL prime western blotting detection reagent (Amersham Biosciences, Uppsala, Sweden), and protein bands were quantified using a LANE 1D system (Sagecreation, Beijing, China).

### H&E staining

A549 xenografts and metastases tissues were collected, fixed in 10% buffered formalin, paraffin-embedded and cut into 4 μm-thick sections, which were then deparaffinized and rehydrated. Hematoxylin and eosin staining was performed using the standard histological technique.

### IF staining

Cells were grown on coverslips, fixed in 4% paraformaldehyde for 10 min and permeabilized with 0.5% TritonX-100 for 10 min. Blocking was performed with 3% bovine serum albumin fraction V (Solarbio, Beijing, China) for 1 h at RT. After washing, the cells were incubated with the primary antibody at 4°C overnight, followed by incubation with a fluorescent-labeled secondary antibody, for 1 h at RT. Cells were then mounted with Antifade Mounting Medium containing DAPI (Beyotime, Shanghai, China). Finally, immunofluorescence signals were visualized and recorded using a Leica SP5II confocal microscope (Leica Microsystems, Mannheim, Germany).

### IHC staining

IHC analysis was performed using the DAKO LSAB kit (DAKO A/S, Glostrup, Denmark). Briefly, tissue sections were deparaffinized, rehydrated and incubated with 3% H_2_O_2_ in methanol for 15 min at RT. The antigen was retrieved at 95°C for20 min using a 0.01 M sodium citrate buffer (pH 6.0). The slides were then incubated with the primary antibody at 4°C overnight. After incubation with the secondary antibody at RT for 1h, immunostaining was developed using DAB, and the slides were counterstained with hematoxylin.

### *In vivo* animal study

Female BALB-C/nude mice were purchased from the Shanghai University of Chinese Medicine (SHUTCM). Animal experiments were approved by the ethics committee of Yanbian University and conducted in accordance with the regulations of the Service of Consumables and Veterinary Affairs-Division of Animal Protection (SCAV-EXPANIM). Five million cells were injected subcutaneously into the dorsal of nude mice. When the xenograft tumor reached 50mm^3^, the mice were randomized into four groups (n=6, per group) as follows: DMSO, BEZ235, TSA, or BEZ235 and TSA. The mice were treated with BEZ235 (30mg/kg/d, p.o.) and TSA (1mg/kg/d, i.p.) alone or in combination. Caliper measurements were performed daily to calculate tumor volumes. For the Kaplan-Meier tumor free survival curves, mice were considered tumor free until tumor volume reached 200mm^3^. The animals were sacrificed 27days after treatment initiation or when the tumor volume reached 2 cm in diameter. The tumors were excised into formalin, and portion of the tumor fresh-frozen in liquid nitrogen for further processing and analysis.

### Metastatic analyses

Female BALB-C/nude mice were intravenously injected with luciferase-labeled A549 cells (0.5×10^6^) via the tail vein. Mice were randomized into four groups (n=5, per group) as follows: DMSO, BEZ235, TSA, or BEZ235 and TSA. The mice were treated with BEZ235 (30mg/kg/d, p.o.) and TSA (1mg/kg/d, i.p.) alone or in combination. Mice were sacrificed 8 weeks after the tail-vein injections. The mice were assessed for metastatic spread to the lymph nodes and to other tissues using an *in vivo* imaging system (IVIS) spectrum system (Perkin Elmer, USA).

### Statistical analysis

Each experiment was performed in triplicate. All data are presented as the mean ± SD. Statistical analysis was performed using the SPSS 17.0 statistical package (SPSS, Inc., Chicago, IL, USA), and comparisons between groups were conducted using One Way ANOVA. Differences were considered statistically significant at *p*<0.05. Tumor free survival was calculated with log-rank (Mantel-Cox) test using GraphPad Prism 5 (GraphPad Software, Inc., San Diego, CA, USA).
